# Wild edible plants of Belarus: from Rostafiński’s questionnaire of 1883 to the present

**DOI:** 10.1186/1746-4269-9-21

**Published:** 2013-04-04

**Authors:** Łukasz Łuczaj, Piotr Köhler, Ewa Pirożnikow, Maja Graniszewska, Andrea Pieroni, Tanya Gervasi

**Affiliations:** 1 Institute of Applied Biotechnology and Basic Sciences, Department of Botany and Biotechnology of Economic Plants, University of Rzeszów, Werynia 502, 36-100 Kolbuszowa, Poland; 2 Institute of Botany, The Jagiellonian University, ul. Kopernika 27,31-501 Kraków, Poland; 3 Department of Botany, Institute of Biology, University of Białystok, ul. Świerkowa 20B, 15-950 Białystok, Poland; 4 Herbarium of the Institute of Botany of the University of Warsaw, University of Warsaw, Al. Ujazdowskie 4, 00-478 Warszawa, Poland; 5 University of Gastronomic Sciences, Piazza Vittorio Emanuele 9, I-12060 Bra/Pollenzo, Cuneo, Italy

**Keywords:** Historical ethnobotany, Wild green vegetables, Wild food plants, Non-timber forest products, Belarus

## Abstract

**Background:**

Belarus is an Eastern European country, which has been little studied ethnobotanically. The aim of the study was to compare largely unpublished 19th century sources with more contemporary data on the use of wild food plants.

**Methods:**

The information on 19th century uses is based on twelve, mainly unpublished, responses to Józef Rostafiński’s questionnaire from 1883, and the newly discovered materials of the ethnographer Michał Federowski, who structured his data according to Rostafiński’s questionnaire and documented it with voucher specimens. Rostafiński’s questionnaire was concerned mainly with Polish territories, but for historical reasons this also encompassed a large part of Belarus, and we analyzed only the twelve responses (out of the few hundred Rostafiński obtained), which concerned the present Belarus. These data were compared with a few 20th century ethnographic sources, and our own 40 interviews and questionnaires from Belarus.

**Results and discussion:**

58 taxa of wild food plants used in the 19th century were identified. Some of them are still used in modern Belarus, others are probably completely forgotten. In the 19th century several species of wild greens were widely used for making soups. Apart from *Rumex*, other wild greens are now either forgotten or rarely used. The list of species used in the 20th and 21st century encompasses 67 taxa. Nearly half of them were mentioned by Rostafiński’s respondents. The list of fruit species has not changed much, although in the 19th century fruits were mainly eaten raw, or with dairy or floury dishes, and now apart from being eaten raw, they are incorporated in sweet dishes like jams or cakes. Modern comparative data also contain several alien species, some of which have escaped from cultivation and are gathered from a semi-wild state, as well as children's snacks, which were probably collected in the 19th century but were not recorded back then.

**Conclusion:**

The responses to Rostafiński from 1883 present extremely valuable historical material as the use of wild food plants in Belarus has since undergone drastic changes, similar to those, which have taken place in other Eastern European countries.

## Background

There has been a renewed interest in wild food plants in recent years due to world-wide concerns about the quality of food made from mass-produced crop plants, which are poor in micronutrients and grown in petroleum based agricultural systems [1-5]. At the same time, old traditions of plant gathering in most countries are being lost and need recording and preserving. This is also relevant for Eastern Europe. Fortunately in this part of the world many nineteenth and early twentieth century studies have managed to capture disappearing plant uses. This is one of the few places in the world where diachronic studies ranging over the period of a century are possible. Over the last few years, reviews of archival ethnographic studies concerning wild food plant use have been published in some eastern and northern European countries: Poland [6-8], Estonia [9], Hungary [10], Sweden [11] and Slovakia [12]. These reviews brought the majority of wild food plant literature and data together, enabling inter-country and inter-region comparisons. It also makes this data available for an international readership, as they were originally, predominantly published in their national languages. However, some eastern European countries still remain *terra incognita* for modern ethnobotany. One of them is Belarus. We have not found any modern ethnobotanical studies concerning this country, apart from a short FAO report on crop genetic resources [13]. At the same time it is a country with a very rich folklore. It was Kazimierz Moszyński (1887–1959), the author of *Kultura ludowa Słowian* (the *Folk culture of Slavs*), who pointed out that the present area of Belarus is one of the parts of Europe where many vestiges of traditional culture had been preserved [14,15]. He made a few expeditions to the Belarusian region of Polesia himself [16] and published an ethnographic monograph of its eastern parts [17]. For many decades, Belarus was treated by Polish ethnographers as one of the most interesting, “archaic” and “backward” places of the former Polish-Lithuanian Commonwealth, ideal for ethnographic research. Even today, due to its political isolation and the fact that a part of its population still lives in traditional-style villages scattered over this heavily wooded country, Belarus is a very important place for European ethnobiology.

Moszyński was not the only Polish ethnographer fascinated with Belarus. Here we should list two other ethnographers: Michał Federowski (1853–1923) and Józef Obrębski (1905–1967). Late 19th century folklore concerning the use of medicinal plants was recorded by the afore-mentioned Federowski in the first volume of his “Lud Białoruski” (“Belarusian Folk”) [18] as well as by one of the leading Polish writers of that time, Eliza Orzeszkowa (1841–1910) [19-28]. What is amazing is that both of them left rich, detailed herbaria documenting the names of plants and their uses. The second and third parts of Federowski’s herbarium are kept in the library of Warsaw University [29] and Orzeszkowa’s main herbarium is stored in Poznań [24-28]. The first part of Federowski’s herbarium was regarded as lost until last year, when it was discovered by one of the co-authors of the article (M.G.). Additional sources of information are the materials gathered by local researcher, Zośka Wieras [30]. Thus we can conclude that the 19th century use of medicinal plants in some parts of Belarus is relatively well documented. Unfortunately, very little information has been published on the use of wild food plants from the same territory [17,31]. At the same time a large and well documented set of observations on the use of wild food plants in 19th century Belarus, made up of responses to Rostafiński’s questionnaire (mainly from 1883), is stored in two Polish botanical institutions, with most data still unpublished [31-36]. As the files of Rostafiński’s questionnaire are some of the most important ethnobotanical documents in Europe, enabling us to draw a detailed picture of the use of wild plants in Belarus at the end of the 19th century, we decided to devote a separate article to them. Our aim was to compare their content with the scattered modern data on wild food plants in Belarus.

## Methods

### Belarus as a study area

The state of Belarus is located in Eastern Europe. It has an area of 207 thousand km^2^ and a population of 9.5 mln (according to the 2012 census). The population density is relatively low (46 people / km^2^). Belarus is a landlocked lowland country with predominantly postglacial landform. Areas of sandy soils are mixed with clays, marshes and peat-bogs. The southern part of the country (Polesia region) is very marshy. A large proportion of the population (ca. two million) lives in the capital city, Minsk. Belarus is located in an area of humid continental climate. The forest vegetation is composed of both coniferous and deciduous species. *Pinus sylvestris*, *Picea abies*, *Alnus glutinosa*, *Betula pendula* and *Quercus robur* are the dominant trees in the heavily wooded landscape (forest cover ca. 40%) [37]. The vascular flora of Belarus contains around 1860 species [38].

Belarus was one of the core parts of the Kievan Rus’. In medieval times it was a part of the Principality of Polotsk, the Grand Duchy of Lithuania, then the Polish–Lithuanian Commonwealth. Later, at the end of the 18th century, through the partitions of the Commonwealth, it became part of the Russian Empire. After World War I, the territory of Belarus was divided between Poland and the Soviet Belarusian Republic (the latter within Soviet Union). In 1939, the Polish part of Belarus was annexed by the Soviet Union and merged with the Soviet Belarusian Republic. After World War II, most of the large Polish minority left Belarus (now it constitutes only 3% of the population). After the collapse of the Soviet Union, it became an independent state in 1991. At the moment the Belarusian nationality dominates in the population, however, two closely related languages are official: Russian and Belarusian, with the former dominating in cities. The main minorities are Russians, Poles and Ukrainians [38-40].

Belarusian cuisine is dominated by potato dishes and bread. Dairy products and pastry dishes (i.e. dumplings) are also eaten on an everyday basis. Soups have been also a major part of dinner. Many dishes are made using fermented ingredients (sourdough bread, sourdough soups, lacto-fermented salads made from cabbage, cucumbers and tomatoes, fermented birch sap etc.) [41,42].

### Characteristics of Rostafiński’s questionnaire

Professor Józef Rostafiński (1850–1928), a Polish botanist from Kraków (Jagiellonian University), composed a 70-question questionnaire concerning all aspects of ethnobotany (traditional cultivated and wild foods, medicine, rituals, dyes etc.). The survey was called “Odezwa do nie botaników o zbieranie ludowych nazw roślin”, which translates as “An appeal to non-botanists to collect folk plant names”. In its largest version it included 70 questions concerning the use of plants, their cultivation, gathering and naming. It was published in 1883 in around 60 Polish language newspapers in Prussia, Austro-Hungary and Russia (at that time Poland was divided into these three empires). Rostafiński received a few hundred responses, which have been partly preserved up to the present and constitute probably the largest ethnobotanical survey of 19th century Europe. The known letters come from the years 1883–1909 (mainly 1883–84). Out of around two hundred authors who wrote to him, most sent him information concerning the contemporary territory of Poland. However, several of them reported the use of plants in the present territory of Belarus and western Ukraine, for historical reasons, as a large proportion of intelligentsia and landowners (typical of Rostafiński’s respondents) in these countries were either Polish or Polonized. In their letters they mostly referred to plants grown or cultivated by peasants, though sometimes they also supplied details on the plants used in manors [31-36].

Responses to Rostafiński were only partly used by their owner and remained, forgotten, in the Jagiellonian University for decades. They were “discovered” at the end of the 20th century on the premises of the Institute of Botany and are stored in the Museum of the Botanic Garden of the Jagiellonian University [31-36]. Twelve of them contain information on the present Belarus and were analyzed in this paper (Table 1). Most of the information contained in them and the original text have not been published, apart from scattered notes on the use of some species (*Heracleum* and *Aegopodium* – [8,36], tree saps – [8,36,43]). The correspondence with one respondent, Maria Twardowska, was characterized in a separate paper [31].

**Table 1:** Characteristics of Rostafiński’s respondents and the location of places they wrote about

**Place for which information was given (the present Belarusian name given in brackets)**	**Region or county**	**Code**	**Surname, First name**	**Biographical information**
Materials stored in the Museum of the Botanic Garden of the Jagiellonian University, ul. Kopernika 27, Kraków				
Nieśwież, Słuck and Mińsk	Mińsk (Minsk)	CZA	Czarnocka, Helena	landowner, sent her letters from Secieszyn (Kleck train stop, Minsk gubernya)
(Nyasvizh, Sluck, Minsk)				
Jeziora (Azyory)	Grodno (Hrodna)	KOR	Korycińska, Aleksandra	no data; wrote her letter from Warsaw, passing on information from her friends
Bobrzyńsk near Bobrujsk (now Babruysk) and surrounding counties	Mohylew (Mohilew)	LAS	Laskarys, Antonina z Zabiełłów	(1835 in Vilnius - ?), landowner [44]
Naliboki	Mińsk (Minsk)	LES	Łęski, Michał	landowner from Chotów
Puków	Ihumeń (Chervyen’)	NAR	Narkiewicz-Jodko, Tomasz	(ok. 1840 -?), landowner (Puków estate in Minsk gubernya)
Pińsk and around (Pinsk)	Pińsk (Pinsk)	ONU	Onufrowicz, Adam	(1856–1914), studied at the Institute of Mining in St. Petersburg and the Technical Academy in Kraków, taught in polytechnics in Austro-Hungary and Russia. He was the main director of a factory in Kysztyma [45]
Kuchcice and Chołuj	Ihumeń (Chervyen’)	OSS	Ossowski, Antoni	no data
Rawonicze	Ihumeń (Chervyen’)	SLO	Słotwińska, J	no data
whole counties	Pińsk, Mozyrz, Rzeczyca (Pinsk, Mazyr, Rechytsa)	SLO	Słotwińska, J	
Weleśnica	Pińsk (Pinsk)	TWA	Twardowska, Maria	(1858–1907), botanist (florist), authored several publications on the flora of the Vilnius region and Polesia [46-48]
n.d.	Pińsk (Pinsk)	NIE	Nielubowicz, W.	landowner
Nowogródek (Navahrudak)	Grodno (Hrodna)	DYB	Dybowski, Władysław	(1838–1910), zoologist and botanist, master of mineralogy (1873), *privatdozent* of general paleontology in the university of Dorpat (now Tartu) (1876); when he responded to Rostafiński he was leasing an estate in Niańków near Nowogródek [49]
Lipów	Rzeczyca (Rechytsa)	WOJ	Woyniłłowiczówna, Jadwiga	no data
Manuscript and herbarium stored in the Herbarium of the Faculty of Biology of the University of Warsaw, Al. Ujazdowskie 4, Warsaw				
whole counties	Wołkowysk, Słonim, Prużany	FED	Federowski, Michał	(1853–1923), amateur ethnographer, the leading researcher of Belarusian verbal and musical folklore; worked on manorial estates, gathered many volumes of data on Belarusian folklore, e.g. [18]; lived in the presen territory of Belarus for most of the time between 1877and World War I; he wrote his answer to Rostafiński’s quest. from Studerowszczyzna
	(Vawkavysk, Slonim, Pruzhany)			
	Vileyka	FED2	Federowski, Michał	

All the letters were written in 1883, apart from Korycińska (1884).

Apart from them, in 2012, another response to Rostafiński’s questionnaire was found by one of the co-authors of this article (M.G.) in the herbarium of the Warsaw University. This never-sent response belongs to Michał Federowski *vel* Fedorowski (an ethnographer working in Belarus mentioned in the Background section of this article). It contains a separate folder with responses to Rostafiński’s questionnaire titled “Rośliny użyteczne u ludu litewskiego okolic Słonima, Wołkowyska i Prużanny. Zeszyt I. Zebrał i opisał Michał Fedorowski” (i.e. “Useful plants of Lithuanian peasants in the area of Słonim, Wołkowysk and Prużanna. Volume I. Collected and described by Michał Fedorowski”), including an appendix describing the use of forest trees in this area and a herbarium documenting the plants mentioned in the text, as well as another file, which is a previously unknown part of Federowski’s herbarium of medicinal plants documenting his chapter in “Lud Białoruski” [18] (“Zioła lecznicze używane przez lud litewski w okolicach Wołkowyska i Słonima z dodatkiem roślin w gusłach i czarach zastosowanie mających. Zeszyt II. Zebrał i opisał Michał Fedorowski”, i.e. “Medicinal herbs used by Lithuanian peasants in the area of Wołkowysk and Słonim with the addition of plants used in superstitions and magic. Volume II. Collected and described by Michał Fedorowski”; the other two parts have been deposited in the library of Warsaw University). “Lud Białoruski”, as its author stated, contains material gathered between 1879 and 1891, but the date of the manuscript is “November 1883” (straight after the publication of Rostafińki’s questionnaire). It is not sure if Federowski ever thought of sending the materials to Rostafiński. It is also possible that he found his questionnaire a useful framework for his own work. This part of the herbarium, concerning non-medicinal uses must have been assembled from the plants he had dried earlier.

Rostafiński’s questionnaire included several questions concerning the use of wild food plants:

“6) Is *ber* [*Setaria italica*] grown? do people gather wild *ber* and other wild grasses, particularly:

7) *manna*, *mielec* [both refer to *Glyceria*], *stokłosa* [*Bromus*], in what quantities, do people use it themselves or bring it to town market?

21) Do people know (at least from tradition) the names *kucmerka* and *słodyczka*? [the question referred to old names of *Sium sisarum*, but yielded answers for the use of *Stachys palustris* and *Polypodium vulgare*, for details see [50].

23) Do local people gather herbs in spring to be used in soups, particularly in famine years, and these herbs are?”.

This is followed by questions 24–33, in which he asks if people know the names of particular plants. These seem like continuations of question 23, as most of the listed plants are green vegetables: *Urtica* (as *pokrzywa*, *żegawka*) and *Glechoma* (as *kurdybanek, bluszczyk*) – no. 25, *Rumex* and *Oxalis* (as *szczaw, zajęcza kapusta*) - no. 26, *Heracleum* (as *barszcz* “plant” – to differentiate it from the soup also called *barszcz*) – no. 27, *Aegopodium podagraria* (as *gir, girz* in no. 28, and *śnitka* in no. 32) and *Artemisia abrotanum* [as *Boże drzewko*] “or other plants fried with butter”. In the responses to these names people usually reported not only local names but also the way these species were eaten.

And further:

“34) Salads and herbs eaten raw, which [plants]?

43) What do the names *wiśnie* [sour cherry], *trześnie* [wild sweet cherry], *czeremchy* [chokecherry] mean in a given place?

44) Is the name *dracz* known for barberry? What berries do people know and under what names: a) raspberry-type composed fruit: pink like raspberry or dark blue, shiny like plums (blackberry), b) currants, red and black with stinking leaves, c) gooseberry, d) strawberry, e) small undershrubs with shiny leaves and round berries, red (*brusznice, kamionki*) or black (*borówki* and *łohynie* vel *pijanice*), f) with more oblong berries[,] cranberries, Cornelian cherry? Or other names?

50) Are nigella, coriander, dill, fennel, anise and caraway cultivated in manor gardens? [although these are mainly cultivated species, the question yielded answers on the use of wild caraway]

58) Do people buy culinary oil or make it themselves? From what? Flax? Cannabis? Poppy (grey or white), sunflower, rape? Or from traditionally used ingredients? [some of the answers concerned wild taxa]”

We also extracted information on wild food plants from the remainder of the letters sent in response to Rostafiński, sometimes in the form of digressions or loose observations.

**Figure 1:**
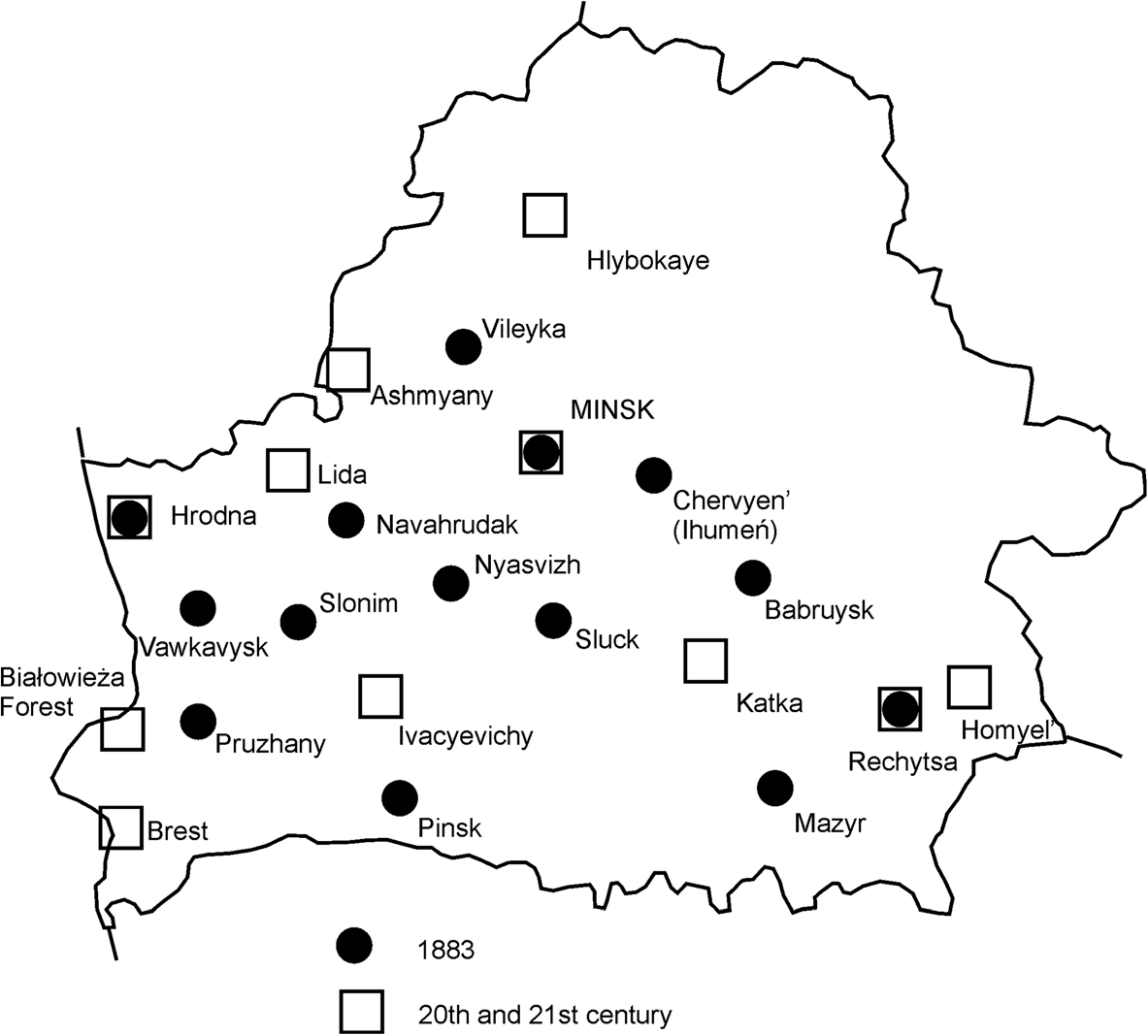
The location of information on plant uses, in most cases county towns, are marked but the information concerns the whole county.

**Figure 2:**
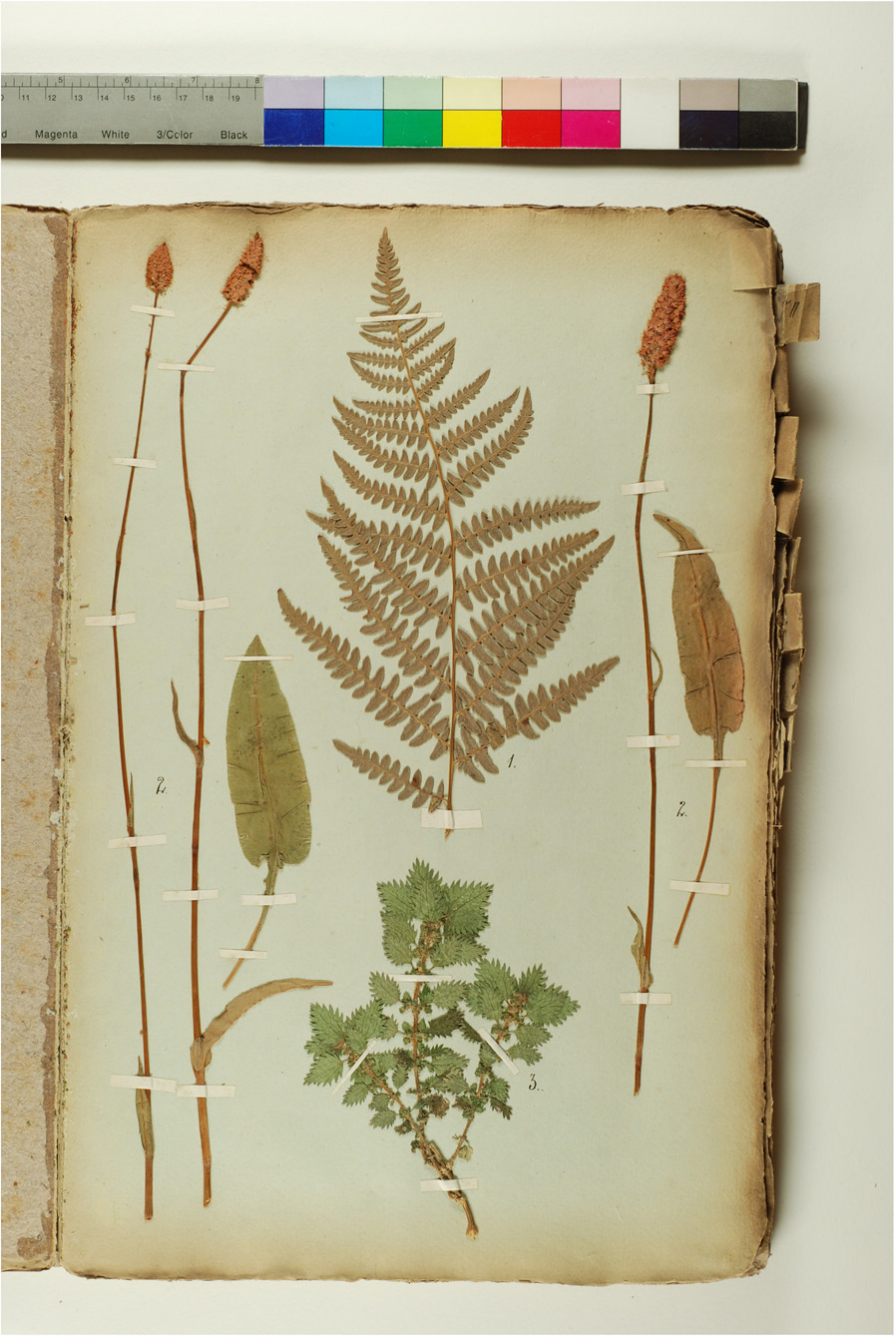
The first page of Federowski’s herbarium.

Thirteen responses to Rostafiński’s questionnaire were analyzed (including the Federowski’s materials). The information contained in them comes from many places in Belarus: from the south and south-east (Polesia), west (Hrodna, Vawkavysk, Pruzhany, Slonim), central part (e.g. Minsk and Ihumen’/Chyerven’), and north (Vileyka) (Figure 1). In many cases the respondents supplied Latin names of the plants they mentioned. Federowski additionally provided voucher specimens for most of the plants he reported (Figure 2). For nearly every plant he also provided a separate note on its use and, separately, the Belarusian peasant name and the name used by the Polish *szlachta zaściankowa* (i.e. poorer mainly Polish landowners of noble origin), who often farmed the land themselves but held Polish aristocratic titles and coats of arms. The north-western part of Belarus which Federowski studied was a nearly equal mixture of these two social and ethnic groups.

### 20th and 21st century data

In order to relate the data from Rostafiński’s questionnaire to the present-day use of plants in Belarus, we tried to gather the scattered data on the use of wild food plants in the 20th and 21st centuries:

•One of the authors (T.G), in 2010 and 2011, interviewed middle-aged and elderly women (aged 45–85) about the use of all food plants (from three locations: the Minsk agglomeration (n = 19), the village of Katka (in the Mohilev region, n = 10) and the village Galyenchitsy (near Ivatsyevichi, in the Brest region, n = 1), as a part of a bachelor’s thesis supervised by another co-author (A.P.), who also took part in some of the interviews.

•The list of plants was extended by interviews and questionnaires supplied by E.P. (n = 10), carried out in 2010–12. This includes five lists of traditional wild food compiled by Belarusian botanists (based on their autobiographical observations from their home places): Dr Oleg Sosinov, associate professor in the Faculty of Botany, University of Hrodna, information from: Kapyl’ county (Minsk region) and Hrodna county, Dr Alla Aleksandrovna Pogodzkaya, Faculty of Pharmacognosy, Vitebsk State Medical University, (info from Homyel’ region), three botanists from the Belarusian Bielovyezhskaya Pushcha National Park; two with E.P.’s elderly family members came from the villages around Hlybokaye (Vitebsk region) and three interviews carried out in the Bryest and Hrodna regions by E.P.’s students of Belarusian origin (carried out with their own elderly family members).

•Information from two 20th century publications containing data on wild food plant use in southern Belarus (Polesia) [17,51-53].

•Retrospectives of settlers who moved from Belarus to Poland after World War II (in the questionnaires of the Polish Ethnographic Atlas from 1948 and 1964–69, for the characteristics of this source see [8]).

•Archives of the Polish ethnographer Adam Fischer stored in the library of the Polish Folklorist Society in Wrocław were searched for material concerning Belarus; five notes on the use of plants were found, three from Narbutowszczyzna near Oszmiana (now Ashmyany), based on herbarium specimens sent by Zofia Koczorowska to Fischer, probably in the 1920-30s, the note on *Acorus* comes from the Lida area and the note on *Prunella* from Antyczkowo, near Oszmiana).

•A Belarusian Internet culinary forums was searched in order to find out which of the listed plants are still a part of everyday culture (not included in Table 2).

•An additional source of comparison (not included in Table 2) was data on plant use in Belarusian villages in NE Poland, adjacent to the border with Belarus, from over two hundred interviews carried out by one of the co-authors (E.P.) [54,55].

**Table 2:** Contemporary use of wild food plants (20-21th century)

	**Modern local name (transliterated from Cyrillic apart from Polish names following Polish orthography (marked PL)**	**Part**	**20th – 21th century use**	**Source**
*Acer platanoides* L.	klyon, yavor	leaves	leaves under baking bread	EP
*Acorus calamus* L.	ayer (also PL), air, babki	leaves, shoot center	leaves under baking bread, formerly; shoots formerly as a spring snack	[51], EP, FS
*Aegopodium podagraria* L.	snitka	leaves	soup	EP
*Allium* sp. ?	PL: dziki czosnek	leaves	spice	EP
*Allium ursinum* L.	cheremsha	leaves	raw	EP
*Aquilegia vulgaris* L.	vodosbor	nectar	raw children’s snack	EP
*Armoracia rusticana* G.Gaertn., B.Mey. & Schreb	khren	roots and leaves	roots – grated into a spicy paste called kren or added to dishes as spice, leaves as spice for fermented sauerkraut, cucumbers and tomatoes, and soups	TG, EP
*Artemisia absinthium* L.	polyn'	leaves	herbal teas	EP
*Berberis vulgaris* L.	n.d.	fruit	raw, snack, juice or salted	EP
*Betula* spp.	byeryoza	sap	fresh and fermented (such drink is called byarozavik)	TG, EP, PAE
		wood shavings	“eaten” (probably added to bread as famine food)	FS
*Carum carvi* L.	tmin, kmin	fruits	spice for bread and sauerkraut	TG, EP
*Chamomilla recutita* (L.) Rauschert	n.d.	inflorescences	herbal tea	EP
*Chenopodium album* L.	labadá (mistakenly as Atriplex), PL: lebioda	leaves	formerly in soups	[51], FS
*Corylus avellana* L.	oryekh, aryekh, aryéshnik, lyeshina,	fruits	mainly raw	[17,51], EP
*Crataegus* sp.	boyáryshnik	fruits (“jablochki”)	fresh and in jams, wine	TG, EP
*Dactylis glomerata* L.	yezha	stalk	inner part as a snack	EP
*Fragaria* spp., mainly *F. vesca* L.	sunítsa, zyemlyanika	fruits	raw, jams, wine etc., formerly also eaten with milk and cream	[17,51], EP
*Glyceria fluitans* (L.) R.Br.	manna, máyna	grains	used until late 1940s to make kasha	[17,52,53], PAE
*Hippophaë rhamnoides* L. *	oblyepikha	fruits	fresh, juice, jams	TG
*Humulus lupulus* L.	khmyel'	fruits	formerly dried, spice for beer and mead and added to bread dough	[17,51], EP
*Juniperus communis* L.	n.d.	pseudo-fruits	raw children’s snack, spice for food and alcoholic beverages	EP
*Lamium album* L.	n.d.	nectar	raw children’s snack	EP
*Linaria vulgaris* L.	l’vinyy zyev	nectar	raw children’s snack	EP
*Lotus corniculatus* L.	miadunka	nectar from flowers	raw children’s snack	EP
*Malus sylvestris* Miller or *Malus domestica* Borkh.	yáblyki	fruits	eaten raw, dried, lactofermented in sauerkraut or boiled	[17,51]
*Malva pusilla* Sm.	yagodki	immature fruits	raw children’s snack	EP
*Nymphaea alba* L.	mákowka (for fruits), húski (for the plant)	seeds	raw as a snack	[17]
*Oxalis acetosella* L.	zayacha kapusta, záyachy shchavyel', zayach’ya kapusta, kislitsa	leaves	raw children’s snack, formerly sometimes used for soups	[51], EP
*Pinus sylvestris* L.	sasná	resin(1), young shoots(2)	raw children’s snack(1), famine food(2)	EP
*Plantago lanceolata* L.	n.d.	leaves	salads	EP
*Poa pratensis* L.	travka	young shoots	raw children’s snack	EP
*Polygonum bistorta* L. (syn. *Bistorta major* S. E. Gray)	PL: wężownik	leaves	eaten with bread during World War I	FS
*Prunella vulgaris* L. (?)	PL: czemborek	aerial parts	infusion drunk as everyday drink	FS
– uncertain identification, the folk name suggests it could also be *Thymus* sp.				
*Prunus cerasifera* Ehrh.*	alychá	fruits	fresh and in jams	TG, EP
*Prunus spinosa* L.	n.d.	fruits	spice for alcohol, raw snack	EP
*Pulmonaria officinalis* L. cf ssp. *obscura* (Dumort.) Murb. (syn. *P. obscura* Dumort.)	myedunitsa	flowers	fresh nectar as a snack and made into herbal teas	TG, EP
*Pyrus pyraster* L.	hróshka, hrúsha, ihrúshka, grush́a lyesnáya	fruits	raw or in boiled dishes, formerly fermented in water and sugar	[17,51], TG
*Quercus robur* L.	dub	leaves and bark	formerly under baking bread	[51], EP
*Ribes nigrum* L.	smoródina chyérnaya	fruits, twig and leaves	fruits – fresh or dried; twigs – decoction; leaves as spice for fermented sauerkraut, cucumbers and tomatoes	TG, EP
*Ribes uva-crispa* L.	kryzhóvnik	fruits	fresh	
*Robinia pseudoacacia* L.	n.d.	flowers	formerly raw snack	EP
*Rosa canina* L. and other spp.	shipóvnik sobáchyy	fruits	fresh and in jams, wine and herbal tea	TG, EP
*Rubus caesius* L.	yezhevika	fruits	raw	EP
*Rubus chamaemorus* L.	struzhýna	fruits	raw	[51]
*Rubus idaeus* L.	malína	fruits	fresh, jam, formerly dried as medicine inducing sweating	[17,51], TG, EP
*Rubus saxatilis* L.	kamyenítsa, kostyanika	fruits	raw	[17], EP
*Rubus* subgenus *Rubus*	azhýna, ozhýna, stryzhýna	fruits	mainly raw due to low abundance, sometimes in wine and hot desserts	[17,51], EP
*Rumex acetosa* L.	shchavyél’, shchavyey	leaves	sour soup called borshch	[17,51], TG, EP, PAE
*Rumex acetosella* L.	verabyóvy shchavyél’	leaves	sour soup called borshch	[51]
*Rumex confertus* Willd.	shchavyey	leaves	soup	EP
*Sambucus nigra* L.	n.d.	fruit	juice, wines, rarely also raw	EP
*Sorbus aucuparia* L.	ryabína, rabina	fruits	mainly jam from frozen fruits, also raw as children’s snack and in herbal infusion or as spice	TG, EP; according to [17] was regarded as poisonous in Polesia
*Sorbus intermedia* (Ehrh.) Pers.	n.d.	fruits	gathered from city greenery in Minsk for preserves	TG
*Stellaria media* (L.) Vill.	zvyezdchátka, makritsa	leaves	fresh in salads, squeezed into juice	TG
*Syringa vulgaris* L. *	siryen’	nectar from flowers	raw children’s snack	EP
*Taraxacum* sp.	n.d.	nectar from flowers, leaves	raw snack, leaves also in salads	EP
*Thymus* spp.	n.d.	flowering tops	herbal infusion, spice for alcohol	EP
*Tilia cordata* Mill.	lípa	flowers, leaves	nectar and leaves as children’s snack, infusion from flowers as beverage	TG, EP
*Trifolium* spp., mainly *T. pratense* L.	klyevyer, trilistnik	nectar from flowers	raw children’s snack	EP
*Urtica dioica* L. and* U. urens* L.	krapiva, PL: pokrzywa	aerial parts	potherb, now rarely; formerly also sour soups	[51], EP, FS
*Vaccinium myrtillus* L.	chernítsa, charnítsa, chyerníka,	fruits and leaves	fruits– fresh, jams and juice, or in milk soups, leaves as spice for fermented sauerkraut, cucumbers and tomatoes; the most widely gathered wild fruits in Belarus	[17,51], TG, EP, PAE
*Vaccinium oxycoccos* L.	klyúkva, klukva, zhuravína	fruits	fresh, jam, kisel, juice, formerly also added to suaerkraut	[17,51], TG, EP. PAE
*Vaccinium uliginosum* L.	lohynya, buyakí, golubika; PL: pijanica	fruits	raw, in many places considered inedible	[51], EP
*Vaccinium vitis-idaea* L.	brushnítsa, brus’nítsa, brusnika	fruits	raw or jam	[17,51], EP, PAE
*Viburnum opulus* L.	kalína	fruits	jams, boiled in *kisyel’*, raw – after drying	[17,51], TG
*Viola tricolor* L.	n.d.	aerial parts	herbal tea	EP

* a non-native cultivated species also collected from the populations escaped from cultivation.

*EP* Ewa Pirożnikow’s questionnaires from Belarusian respondents (mainly botanists); *TG* Tanya Gervasi’s field interviews; *PAE* Data from the Polish Ethnographic Atlas – retrospectives of Poles resettled from Belarus in the 1940s; *FS* Adam Fischer’s archives (Polish Folklorist Society, Wrocław).

The contemporary data are presented using transliteration of the Cyrillic alphabet for Belarusian/Russian names and using the official Polish orthography for Polish names. Belarusian plant names and contemporary geographical names were Romanized using the BGN/PCGN standard used by the United States Board on Geographic Names and by the Permanent Committee on Geographical Names for British Official Use [56].

## Results

Fifty-eight identified botanical species were mentioned altogether by Rostafiński’s respondents from Belarus. Five taxa remained unidentified (Table 3). The largest category is composed of the green parts of plants (27 species), which were usually consumed in the form of soups (23 species). Two kinds of soups were distinguished: sour (*kisla vara*) and non-sour (*presna vara*). The first was made by leaving the ingredients to ferment for a few days or by adding acidic ingredients. Sorrel (*Rumex* spp.)*,* ground elder (*Aegopodium podagraria*) and hogweed (*Heracleum sphondylium* s.l.) were used in sour soups. Nettle (*Urtica* spp.) and fat hen (*Chenopodium album*) were, according to Federowski, made into non-sour soups, though other data suggest that sometimes they occurred in sour soups as well and that this division is not very sharp. Sorrel was sour by itself, whereas hogweed was probably lacto-fermented. Soured birch and maple sap were also added to wild vegetable soups (e.g. with ground elder) to make them sour. According to Federowski one of the wild vegetables were commonly dried for winter use. Apart from the wild plants his herbarium also contains two cultivated species (thus not included in Table 2) eaten raw as salads: *Borago officinalis* L. (voucher specimen 21; *hurecznik, ahurecznik/ogórecznik* eaten with cream and salt) and *Tropaeolum majus* L. (22; *nastulek, nastulka,* flowers eaten with cultivated lettuce). He also mentions the raw use of some wild lettuce called *zieziulina sałata*, but does not provide a specimen.

**Table 3:** Wild food plants used in Belarus according to Rostafiński’s respondents

**Scientific name**	**Rel.**	**Local name in 19th c.**	**Part used**	**Use**	**Vo.**	Letter****
		**Names used by Poles marked with PL**				
*Acer platanoides* L.	H	klon	sap	fresh or added to sour soups	50	FED, WOJ
*Acorus calamus* L.	L	ajer, tatarak	leaves	leaves added under baking bread (which was supposed to have carminative properties as well)		LAS
*Aegopodium podagraria* L.	H	śnitka, snitka, sznitka, śniatka, PL: śnitka	aerial parts	soups, sour and non-sour, potherb, rarely dried for winter use	4	CZA, DYB, FED, KOR, LAS, LES, NAR, OSS, SLO, TWA, WOJ
*Allium* sp. ?	N	PL: czosnek dziki	n.d.	n.d.		LAS
*Anchusa arvensis* (L.) M.Bieb.	H	padasocik, PL: podosocik	aerial parts	non-sour soups	11	FED
*Anethum graveolens* L. (?)	N	kopr	aerial parts	spice		LES
*Armoracia rusticana* G.Gaertn., B.Mey. & Schreb	O	chrin, PL: chrzan	n.d.	so obvious that use not given, only one author wrote about salad with vinegar		(LAS, LES, NAR, NIE, OSS, TWA,WOJ)
*Berberis vulgaris* L.	N	berberys (also PL)	fruits	raw		FED
*Betula* spp.	H	bereza, bieroza, PL: brzoza	sap	fresh, added to sour soups, fermented into a refreshing drink with dried apples, bread rinds and oak bark, or sugar	45	FED, NIE, WOJ
*Carum carvi* L.	L	kmin	fruits	spice for bread, sauerkraut, fresh cheese, spirits		CZA, DYB, FED, OSS, SLO, WOJ, (LAS, ONU)
*Centaurea cyanus* L.	H	wałoszka, PL: wołoszka, włoszka, bławatki	very young shoots	non-sour soups	9	FED, KOR
*Chenopodium album* L.	H	lebieda, PL: lebioda	aerial parts	non-sour soups, dried for winter use	14	FED, KOR, CZA, ONU, OSS, WOJ, (TWA)
*Cirisum arvense* (L.) Scop.	H	asot, PL: oset	aerial parts	non-sour soups	10	FED
*Convolvulus arvenis* L.	H	bierozka,PL: bierozka, brzózka	aerial parts	non-sour soups	7	FED
*Corylus avellana* L.	H	harieszyna; PL: orzechy, orzeszyna, leszczyna	fruits	n.d.	48	FED, LAS
*Fallopia convolvulus* (L.) Á.Löve	H	podbierozka (also PL)	aerial parts	non-sour soups	8	FED
*Fragaria* spp., mainly *F. vesca* L.	H	sunicy, paziomki, poziemi, sunyca, PL: poziomki	fruits	raw	26	FED, OSS, (LAS, NIE, WOJ)
*Glyceria fluitans* (L.) R.Br. and most likely other related *Glyceria* species	L	manna (also PL), majńo	grains	made into groat (*kasha*), mainly boiled in milk, sold in the market of Pińsk, in many places stopped being used in the 1850-60s		FED, LAS, NIE, ONU, OSS, SLO, TWA, WOJ
(in the letters as *Glyceria* and *Glyceria fluitans*)						
*Helianthus tuberosus* L.*	N/D	bulba, bulwa	bulbs	roasted or fried, mainly planted but one report says that it was very persistent and ”self-sowing”		(DYB, NAR, ONU, TWA, WOJ)
*Heracleum sphondylium* L.	H	borszcz, barszcz, barszczewnik, PL: borszcz, barszcz		sour soups, potherb, often dried for winter	19	CZA, DYB, FED, OSS, SLO, TWA, (LAS)
*Lamium* sp. or *Pulmonaria* sp.?	N	miedunka	aerial parts	potherb		CZA
*Nymphaea alba* L.	L	[grzybienie]	seeds	n.d.		NIE
*Oxalis acetosella* L.	H	zazulin szczawiej, PL: szczaw kukawki, szczaw zajęczy	leaves	sour soups	17	FED, (WOY)
*Papaver somniferum* L.*	L	samosiejka, widuk	seeds	“for ordinary use ”, i.e. bread and sweets	-	DYB
*Plantago major* L.	H	babka (also PL)	leaves	non-sour soups	6	FED2
*Plantago* sp.	L	tryputnik	leaves	sour soups, potherb		OSS, SLO
*Polygonum aviculare* L. or *Plantago major* L. ?	N	podorożnik	aerial parts	sour soup		OSS
*Polygonum bistorta* L. (syn. *Bistorta major* S. E. Gray)	H	oborocień, obaracień, oberek, ober, PL: ober	leaves, seeds	sour soup, seeds for flatbread, particularly in the 1855 famine	2	DYB, FED
*Prunus padus* L.	O	czeremcha czeremszyna, czeromcha, PL: czeremcha	fruits	n.d.	23**	FED
*Prunus spinosa* L.	L	PL: tarnina	fruits	n.d.		(LAS)
*Pteridium aquilinum* (L.) Kuhn	H	paporocień, paporotnik, PL: paproć	rhizomes	dried, ground and mixed with ordinary flour to make bread	1	FED2
*Pyrus pyraster* L.	L	PL: gruszki dziczki	fruits	raw and conserved		ONU
*Quercus robur* L.	O	dub	leaves	under baking bread (added also due to its carminative properties as well)		LAS
*Ranunculus repens* L.	H	padśnitnik, PL: podśnitnik		non-sour soups	5	FED
*Raphanus raphanistrum* L.	A	swierzepa, sweripa, świrzepa	leaves	soups, potherb		NIE, TWA
*Ribes nigrum* L.	N	smrodziny, smorodinka, smrodyna, PL: smrodziny, czarne porzeczki	fruits, leaves	fruits raw, leaves used in manors to make a fizzy drink and to make vodka taste “older” (NIE)		FED, NIE, WOJ, (LAS)
*Ribes spicatum* Robson	N/R	parieczki, PL: porzeczki	fruits	raw		FED
*Ribes uva-crispa* L.	L	jagrest, PL: agrest	fruits	raw		FED
*Rosa* sp.	O	PL: róża	flowers	formerly fried in batter, in a Polish manor, rare		SLO
*Rubus idaeus* L.	O	maliny (also PL), małyna	fruits	raw		FED, OSS, WOJ, (LAS, NIE)
*Rubus nessensis* W.Hall	D/R	jaryna	fruits	raw		WOJ
*Rubus saxatilis* L.	H	kościanicy, PL: kościanki	fruits	raw	28	FED
*Rubus* subgenus *Rubus* (probably mainly *Rubus plicatus* L.*, R. nessensis* W.Hall and *R. caesius* L.)	H	ażyny, czornyje maliny, ożyna, PL: ożyny	fruits	raw	24	FED, WOJ, (NIE, LAS)
*Rumex acetosa* L.	H	szczawiej, PL: szczaw	leaves	sour soups	16	FED
*Rumex crispus* L.	H	karpacz (also PL)	leaves	sour soups	20	FED
*Rumex* sp. (probably mainly *R. acetosa* L.)		szczaw, szczawel	leaves and stalks	raw and in sour soups, as OSS put: “with kvas, whey, sweet or sour milk, butter milk, cream or pig fat (slonina) with vinegar”		NIE, OSS, ONU, SLO, (CZA)
*Rumex thyrsiflorus* Fing.	H	harabiniec, PL: szczaw polny	leaves	sour soups	18	FED
*Sambucus nigra* L.	N	PL: bez	flowers	formerly fried in batter, in a Polish manor, rare		SLO
*Scirpus lacustris* L.	L	[sitowie]	young stalks	raw		NIE
*Silene vulgaris* (Moecnch)Garcke	H	laskouka,PL: laskówka	aerial parts	non-sour soups, dried for winter use	12	FED
*Silene vulgaris* (?)	N	łuskawka	aerial parts	potherb		KOR
*Sinapis arvensis* L.	H	redźkouka, świerepa, PL: świrepa, świerżop		non-sour soups, dried for winter use	15	FED
*Sinapis arvensis* L. or *Raphanus raphanistrum* L.	N	świrzepa	leaves	soup or with kasha		ONU, (TWA)
*Sorbus aucuparia* L.	N	PL: jarzębina	fruits	raw after freezing		CZA
*Stellaria media* (L.) Vill.	H	makryca bieła, PL: mokrzyca biała	aerial parts	non-sour soups, dried for winter use	62***	FED
*Trapa natans* L.	L	PL: orzechy wodne	seeds	raw and boiled		NIE
*Urtica* spp. (3 records confirm *U. dioica* L. and 4 rec. – *U. urens* L.)	H	krapiwa, kropiwa, kropywa, życzka, żyszka, żyżka, prokywa, prokiwa, pokrzywka, krapiwa piekuszcza, U. urens also rzeszka, rzyczkaja krapiwa; PL: rzeszka, pokrzywa, U. urens also: żagawka, żegawka, rzeszka	aerial parts	non-sour and sour soups	U. urens - 3	CZA, DYB, FED, LAS, LES, NAR, ONU, OSS, TWA, WOJ, (SLO)
*Vaccinium myrtillus* L.	L/O	czarnica, czernyca, czernica, czarnicy, czornyje jahody, czerniec; PL: czernice, czarne jagody	fruits	raw, commonly dried; also boiled with milk into a kind of soup	27	DYB, FED, OSS, NIE, WOJ, (LAS)
*Vaccinium oxycoccos* L.	L/O	żurauliny, żurachwyna, PL: żurawiny, żórawiny	fruits	raw, stored for months in water, in manors made into kissel (with potato flour)		DYB, FED, OSS, NIE, WOJ, (LAS)
*Vaccinium uliginosum* L.	L/O	durnicy, bałabony, łochwaczi, PL: durnice	fruits	raw		FED, FED2, NIE, (LAS), whereas WOJ says that it is narcotic
*Vaccinium vitis-idaea* L.	L/O	brusznicy (also PL), bruśnica, brusznyca	fruits	raw, in manors made into jams and pickles	25	FED, OSS, WOJ, (DYB, LAS, NIE)
*Veronica* cf *persica* Poiret	H	makryca bieła, PL: mokrzyca biała	aerial parts	non-sour soups, dried for winter use	13, 62***	FED
*Viola* cf *odorata* L.	N	PL: fiołki	flowers	formerly fried in batter, in a Polish manor, rare		SLO
?		mirsik	aerial parts	soups		SLO
?		mrzyk	aerial parts	soups		SLO
?		opich	aerial parts	soups		SLO
?		saładucha, smaktucha, PL: sołoducha	aerial parts	non-sour soups		FED
?		zieziulina sałata	aerial parts	raw as salad (like lettuce)		FED

*Rel*. Reliability of identification (after [2]): *H* Confirmed by voucher specimen; *O* Obvious common name universally used over a large area; *L* Probable Latin name or scientific name used in the language of a given country, given by a non-botanist; *N* Determined using comparative analysis of folk names; *R* determined with the help of the data of a species range or/and habitat; *D* determined with the help of the verbal description.

* A non-native cultivated species also collected from the populations escaped from cultivation.

** The voucher specimen belongs to *Euonymus europaea* L., an obvious mistake.

*** The name *makryca bieła* seems to refer to both *Stellaria media* and *Veronica* cf *persica* Poiret. Although in the part of Federowski’s herbarium devoted to edible plants only *Veronica* occurs (no. 13), in the medicinal herbarium both taxa are put together under the same name (no. 62). As the two species often grow together and have a mild taste, and bearing in mind that *Stellaria media* is still called *makryca* in Belarus, we infer that both taxa were probably used.

**** Letter codes are created from the first three letters of the author’s name, full names are given in Table 1. Author codes given in brackets mean that the species was mentioned but it is not clear if it was used as food.

*Vo* Voucher specimen number given by Federowski.

The second largest category, 18 species, was made up of fleshy fruits. Fruits in 19th century Belarus were mainly eaten raw, as a few of Rostafiński’s respondents pointed out. Sometimes they were incorporated into dishes with milk or dough (soup, dumplings, *kisiel/kisyel’*). Only manors prepared more sophisticated wild fruit desserts containing sugar.

A few spices are also mentioned, such as the fruits of *Carum carvi,* roots of *Armoracia rusticana* or aromatic leaves put under baking bread (*Quercus robur, Acorus calamus*).

No underground wild plant organs were gathered apart from the already mentioned horseradish and bracken (*Pteridium aquilinum*) rhizomes used only in the Vileyka region: during bad harvest years dried bracken was pounded in a wooden mortar and mixed with rye flour into ordinary grain dishes.

Out of the species in the 19th century only 32 species have been reported as used in 20th or 21st century studies. In these modern studies, however, new plant taxa are reported as used, mainly children’s snacks or alien species of fruits. Altogether, we collected data on the culinary use of 67 taxa in the 20th and 21st centuries (Table 2, 3), which gives 93 edible taxa recorded at some point between 1883 and the present.

## Discussion

**Figure 3:**
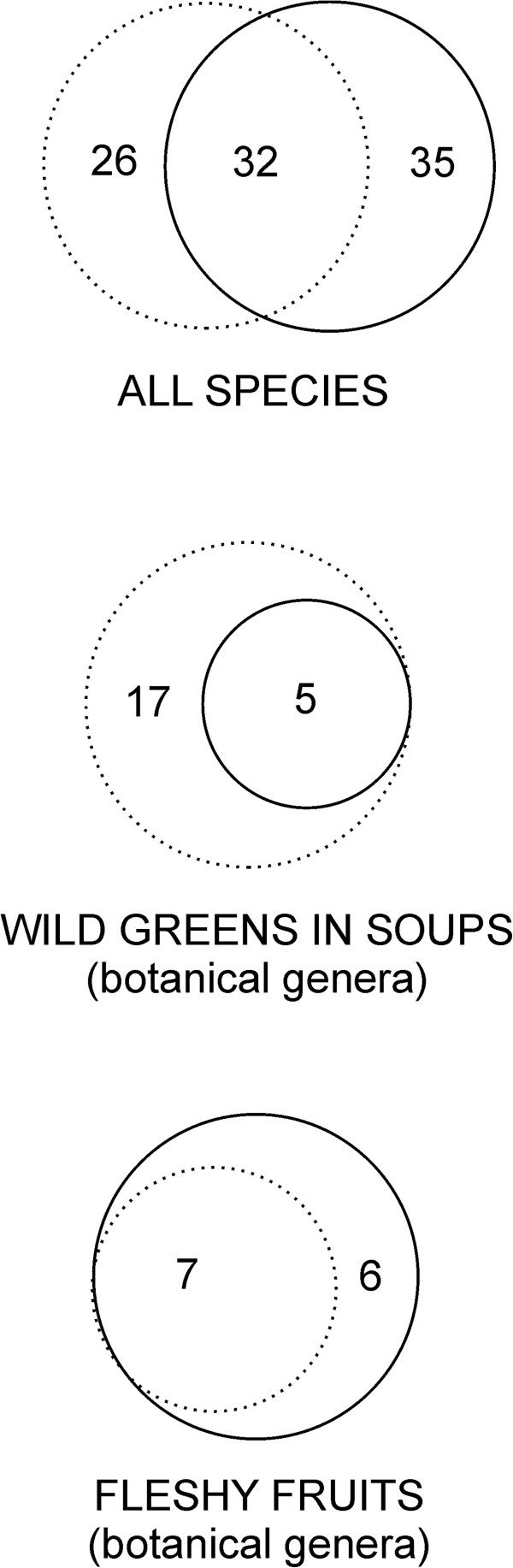
The relationships between the taxa recorded in Rostafiński’s questionnaire from 1883 (dotted line) and the 20th and 21st century data (solid line): while the number of wild greens genera used in soups decreased, the number of wild fruit genera used in nutrition increased.

We can assume that only some of the plants used in Belarus in the 19th century are used nowadays. For example, using *Pteridium aquilinum* rhizomes and *Polygonum bistorta* seeds as staples was already a curiosity by the end of the 19th century. Also the reported wide use of wild green vegetables has immensely decreased in Belarus, with the exception of the use of *Rumex* spp. to make a kind of sour soup, still an important part of Belarusian cuisine. Thus, a process similar to other eastern European countries has occurred in Belarus, where the use of most wild greens has ceased [6-12,57] and the main edible plants used or remembered are fruits and children’s snacks (compare [6,58]). Also, in contemporary internet culinary forums in Belarus, fruits are the dominant wild food category mentioned. The use of wild vegetables has decreased (Figure 3) in a similar manner among the Belarusian minority in NE Poland investigated by one of the co-authors of this article (E.P.), where many of the plants listed in this study are remembered mainly as old-time poverty food (e.g. *Urtica* spp.*, Chenopodium album, Aegopodium podagraria* etc.).

Pietkiewicz, in his 1928 ethnographic monograph of a part of Polesia, mentioned only *Urtica, Rumex, Oxalis* and *Atriplex* (mistakenly for *Chenopodium*) as wild green vegetables used in food [51], whereas Moszyński, also in 1928 [17] did not even mention the use of any other greens apart from *Rumex,* although Rostafiński’s respondents, only 40 years before, mentioned more species used in that area.

The resilience of sorrel in Belarusian and other north and eastern European cuisines [6-12], i.e. the fact that it is still widely used, while other wild greens have declined so much, is mysterious. The most likely explanation is that it is appreciated due to its sour taste and smooth texture. The sour taste has been highly appreciated in traditional eastern European cuisines and most sour foods are produced by lactic fermentation of pickling. Thus sorrel’s sour taste may have automatically placed it in the human realm of transformed food, even before it was cooked. Paraphrasing Lévi-Strauss’s division [59], instead of Raw versus Cooked (in the French original *cru* and *cuit*), we could say Raw versus Cooked/Sour. By belonging to the Sour/Cooked domain, sorrel was singled out from other wild vegetables.

The resilience of the contemporary use of wild fruits such as *Rubus idaeus, Vaccinium* spp. and *Viburnum opulus* in Belarus may be explained by cultural attachment to these species, which are perceived as “very healthy”. Selling *Viburnum opulus* fruits, for example, at the open markets of Minsk in September is still very popular, and most of the sellers and customers ascribed to the berries – which are sometime also consumed raw, as snacks – important preventative properties. In the 19th century *Viburnum* was not even reported as wild food, but rather as medicine. This reinforces the findings that the permanence of Traditional Plant Knowledge in the context of cultural changes may be directly related to the success of certain food plants, which are perceived as *food-medicines*, as, for example, other contemporary field ethnobotanical studies among migrants have demonstrated [60,61].

The differences between the species eaten in the 19th century (Table 2) and contemporary uses (Table 3) do not only arise from the fact that the use of some species has ceased. Some species listed nowadays may have been used in the 19th century but the amount of observations (only from 13 respondents) was not enough to capture it. Also, the structure of Rostafiński’s questionnaire was very specific, he pre-suggested certain taxa, which he was particularly interested in, which may have slightly biased the information given. Children’s snacks reported in the contemporary data must have been collected in the 19th century, but were not recorded then. A similar situation occurred in Poland, when a questionnaire among botanists revealed a long list of minor children’s snacks, never noted before [62].

The commonness of drying wild vegetables for winter use in soups, observed by Federowski, is worth noting. Drying wild greens for human use as a preservation technique occurs in parts of China (see e.g. [63]) but has not been observed in Europe. The fact that these plants were preserved suggests that they had a high cultural status. It is even more puzzling, then, that their presence in contemporary Belarusian cuisine is so reduced.

Of course, it is possible that more in-depth studies in rural Belarus would confirm the survival of some uses. Moreover, a noteworthy phenomenon is the gathering of several recently established non-native taxa. Edible fruits such as *Hippophaë rhamnoides, Prunus cerasifera* and *Sorbus intermedia,* which are cultivated, are also gathered from wild locations (i. e. as garden escapees) or from populations planted as a part of town greenery. Also *Rumex confertus* is a recently spreading large-leaved sorrel species incorporated into cuisine, like native *Rumex* species.

As the use of wild vegetables was more common in Belarus in the 19th century than in Poland as observed by Moszyński, Federowski and a few other ethnographers, we can assume that even now we may find more vestiges of traditional plant use in this country, making it a promising arena for future ethnobotanical studies. Here we point out just a few of the ethnobotanical phenomena in Belarus which should be studied in detail:

•The strong tradition of fermented dishes made from both cultivated plants like cucumbers, cabbage or tomatoes, as well as wild ingredients, e.g. mushrooms (mainly *Lactarius* spp.), birch sap or wild plants used as spices for fermentation (e.g. *Quercus robur* leaves, *Ribes nigrum* leaves);

•The role of tree sap in traditional culture – as Belarus is the only country in Europe where the collection of tree sap is regulated by the state [43] and is extremely popular there;

•The culinary use of marsh and water plants in the wetlands of Polesia (in the 19th century the use of *Scirpus lacustris, Trapa natans* and *Nymphea alba* was recorded in this area);

•The level of preservation of the use of wild green vegetables.

The recorded wild food plant taxa constitute 5% of the country’s flora. It is lower than for Hungary – 7% [10], Estonia – 6% [9] and Poland – 5.5% [8] but higher than Slovakia – 3.5% [12]. However, the amount of data available from Belarus is lower than from the first three countries, which means that several taxa with minor importance in traditional nutrition could be yet to be discovered. The general structure of various use categories and the sequence of their disappearance from the contemporary diet, as well as some culinary vogues (like jam- making, in the 20th century) are astoundingly similar to those reported from other northern and eastern European countries [5-12,58].

## Conclusions

The responses to Rostafiński’s questionnaire from 1883 present extremely valuable historical material as the use of wild food plants in Belarus has since undergone drastic changes, similar to those, which have taken place in other Eastern European countries. Although most taxa reported in this study have been used in other Slavic countries, the local food culture preserved, at least up to the early 20th century, many archaic features, e.g. the wide use of lacto-fermented wild food plants, drying wild vegetables for winter etc. Further studies on the level of preservation of the uses of plants reported by Rostafiński’s respondents are needed.

## Competing interests

The author’s declare that they have no competing interest.

## Authors’ contributions

All the authors took part in writing the paper, additionally ŁŁ: initiated and coordinated the study and wrote the first version of the paper; PK: analyzed the history of Rostafiński’s questionnaire and, together with ŁŁ, analyzed its content; MG: found Federowski’s questionnaires and provided literature on Federowski’s studies; EP, TG and AP provided field data on contemporary plant use in Belarus. All authors read and approved the final manuscript.
